# A New Triterpenoid Saponin from *Abrus precatorius* Linn

**DOI:** 10.3390/molecules17010295

**Published:** 2011-12-30

**Authors:** Zhi-Hui Xiao, Fa-Zuo Wang, Ai-Jun Sun, Chuan-Rong Li, Cai-Guo Huang, Si Zhang

**Affiliations:** 1 Key Laboratory of Marine Bio-Resources Sustainable Utilization, South China Sea Institute of Oceanology, Chinese Academy of Sciences, Guangzhou 510301, China; Email: xzh_77@yahoo.com.cn (Z.-H.X.); wangfazuo@scsio.ac.cn (F.-Z.W.); sunaj@scsio.ac.cn (A.-J.S.); llchuanr@163.com (C.-R.L.); 2 Graduate University of Chinese Academy of Sciences, 19 Yuquan Road, Beijing 100049, China; 3 Department of Biochemistry and Molecular Biology, College of Basic Medical Sciences, Second Military Medical University, Shanghai 200433, China; Email: huangcaig@hotmail.com

**Keywords:** *Abrus precarorius* Linn, triterpenoid, saponin, cytotoxicity

## Abstract

A new triterpenoid saponin, 3-O-*β*-D-glucopyranosyl-(1→2)-*β*-D-glucopyranosyl subprogenin D (**1**), together with six known triterpenoids: subprogenin D (**2**), abrusgenic acid (**3**), triptotriterpenic acid B (**4)**, abruslactone A (**5**), abrusogenin (**6**) and abrusoside C (**7**) were isolated from the leaves and stems of *Abrus precatorius*. Their structures were elucidated on the basis of physical and NMR analysis, respectively. Compounds **5** and **6** showed moderate cytotoxicity against MCF-7, SW1990, Hela, and Du-145 cell lines. Compounds **1**, **2** and **4** were isolated from this plant for the first time.

## 1. Introduction

*Abrus precatorius* Linn belongs to the family Leguminosae. Its seeds, known as Xiang-si-zi, have been used in China as an insecticide and for treatment of some skin diseases since ancient times [1]. Besides, the leaves and roots are sweetish and traditionally used to cure fever, stomatitis, asthma and bronchititis [2]. Several groups of biologically active secondary compounds including alkaloids [3], flavones [4], triterpenoids [5] and isoflavano-quinones [6] have been isolated from this plant, some of which possess anti-inflammatory [7], antibiosis [8], antiplatelet [9], and anti-implantation [10] properties. In our research on bioactive compounds from *Abrus precatorius* collected from the mangrove wetlands of Hainan Island, China, a new 3-O-*β*-D-glucopyranosyl-(1→2)-*β*-D-glucopyranosyl subprogenin D (**1**), as well as six known ones (compounds **2**–**7**) were obtained. The structure of the new compound was elucidated using 1D, 2D NMR and MS experiments, while the configuration of **1** was defined by NOESY spectroscopy. Compounds **2**–**7** were identified as subprogenin D (**2**) [11], abrusgenic acid (**3**) [12], triptotriterpenic acid B (**4**) [13], abruslactone A (**5**) [14], abrusogenin (**6**) [15] and abrusoside C (**7**) [15], respectively, by comparison of their spectroscopic data with those reported in the literature. Compounds **1**, **2** and **4** were isolated from this plant for the first time.

## 2. Results and Discussion

**Figure 1:**
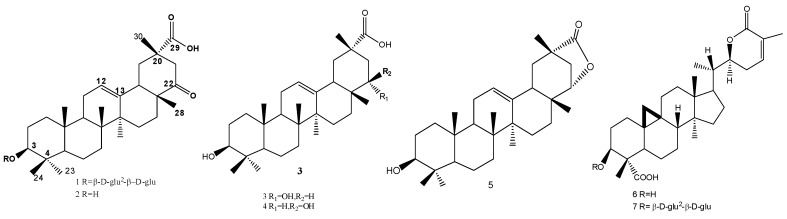
Structures of compounds **1**–**7**.

The aqueous EtOH extract of the *Abrus precatorius* was suspended in water, and then partitioned with petroleum ether, EtOAc, and *n*-butanol by liquid-liquid extraction respectively. The EtOAc and *n*-butanol fractions were successively subjected to repeated silica gel column, Sephadex LH-20 to yield compounds **1**–**7** (Figure 1).

**Figure 2:**
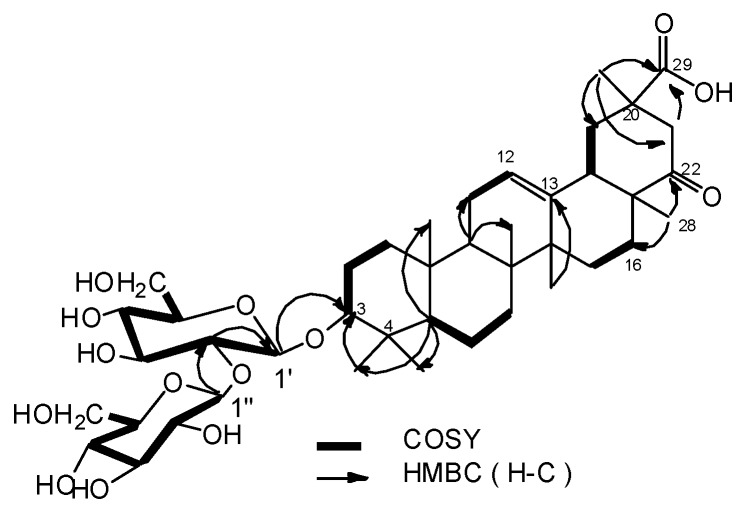
Key HMBC and COSY correlations of **1**.

**Figure 3:**
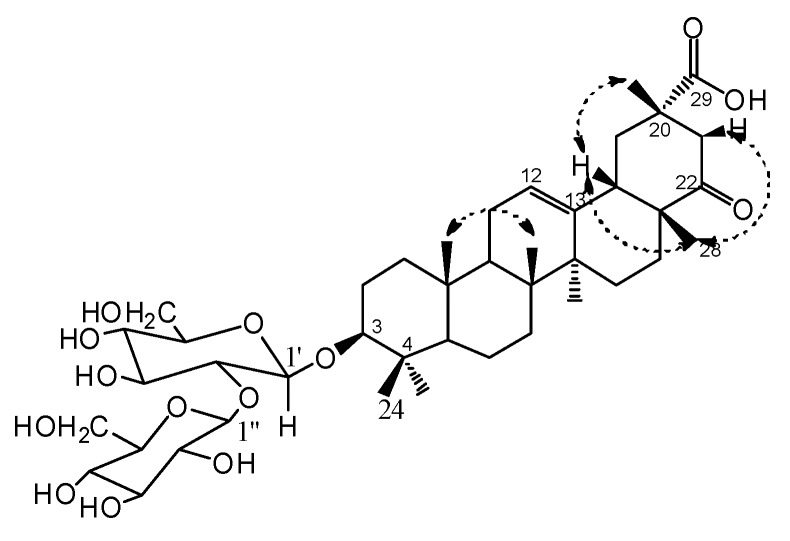
Key NOESY correlations of **1**.

Compound **1** had the molecular formula C_42_H_66_O_14_ as deduced from HRESI-MS *m/z* 817.4345 [M+Na]^+^ (calcd for C_42_H_66_O_14_Na, 817.4350) and NMR data (Table 1). The IR spectrum exhibited absorption bands at 3458 (OH), 1727(C=O), 1693(C=O) and 1624 (C=C) cm^–1^. Seven methyl groups (δ_H_ 1.42, 1.32, 1.27, 1.23, 1.14, 0.91 and 0.86), one oxygenated methine proton (δ_H_ 3.29, dd, *J* = 5.0, 11.0 Hz), and one olefinic proton (δ_H_ 5.31, br s) were observed in the ^1^H-NMR spectrum. The ^13^C-NMR and DEPT data confirmed the presence of seven methyl carbons (δ_C_ 28.1, 25.5, 21.6, 20.9, 16.8, 16.7, 15.6), two olefinic carbons (δ_C_ 124.7, 141.4), one oxygenated methine carbons (δ_C_ 89.0), a carbonyl carbon (δ_C_ 214.9) and a carboxylic carbon (δ_C_ 178.6) (Table 1). The ^1^H- and ^13^C-NMR spectra of **1** has the characteristic of Δ^12^ oleanene skeleton [16]. Comparison the ^13^C-NMR data of **1** with **2** except for the C-3 signal (δ_C_ 89.0) which shifted down field by 11 ppm, others were in accordance with that of 3*β*-hydroxy-22-oxo-12-oleanen-29-oic acid (**2**) [11]. The locations of carbonyl carbon and carboxylic carbon could be confirmed by the HMBC correlations from δ_H_ 1.23 (Me-28) to δ_C_ 214.9, 26.6 (C-16), and δ_H_ 1.42 (Me-30) to δ_C_ 178.6, 41.6 (C-19), 46.5 (C-21) (Figure 2). Moreover, the ^1^H and ^13^C-NMR spectra of **1** showed two sugar anomeric protons at δ_H_ 5.26 (1H, d, *J* = 7.0 Hz) and δ_H_ 5.03 (1H, d, *J* = 7.0 Hz) and carbons at δ_C_ 107.2 and 105.3 (Table 1). The monosaccharides were analysed as *β*-D-glucose with acetylated alditols derivatives by GC using authentic samples as references after hydrolysis of **1**. This also could be validated by a combination of the coupling constants (*J* = 7.0 Hz for H-1″ and *J* = 7.0 Hz for H-1′) and 1D, 2D-NMR experiments. The signal at δ_C_ 89.0 in the ^13^C-NMR suggesting that the *β*-D-glucose moieties are linked to the oxygen at C-3 of the aglycon [17].This deduction and sequence of inter-glycosidic linkages were deduced from the following HMBC correlations: H-1′ (δ_H_ 5.03) of inner glucose with C-3 (δ_C_ 89.0) of sapogenin, H-1′′ (δ_H_ 5.26) of terminal glucose with C-2′ (δ_C_ 83.9) (Figure 2). The relative configuration of the hydroxylated carbon (C-3) was assigned as *β* form mainly on the basis of ^1^H-NMR coupling (1H, dd, *J* = 5.0, 11.0 Hz, H-3) [17] and by comparison with **2**. In NOESY spectrum, the key NOE correlations of H-28/H-21β, H-28/H-18 and H-30/H-18, showed that H-30, H-28, H-21β, H-18 were on the same face, so the relative stereochemistry were determined (Figure 3).

**Table 1:** ^1^H- (500 MHz) and ^13^C-NMR (125 MHz) data of compound **1** (in Pyr-d_5_, *δ* in ppm, *J* in Hz).

No.	δ_C_	δ_H_	Key HMBC (H to C)
1	38.7 CH_2_	1.40 (1H, m, H-1a)0.82 (1H, m, H-1b)	C-2,C-3
2	27.3 CH_2_	2.24 (2H, m, H-2)	C-1,C-3
3	89.0 CH	3.29 (1H, dd, *J* = 5.0, 11.0 Hz, H-3)	C-1′, 23, 24
4	39.5 qC		
5	55.6 CH	0.71 (1H, br d, *J* = 11.5 Hz, H-5)	C-23,24,25
6	18.4 CH_2_	1.64 (1H, m, H-6a),	C-24
		1.46 (1H, m, H-6b)	
7	32.8 CH_2_	1.48 (1H, m, H-7a),	
		1.29 (1H, m, H-7b)	
8	39.9 qC		
9	47.6 CH	1.56 (1H, m, H-9)	
10	36.8 qC		
11	23.8 CH_2_	1.84 (2H, m, H-11)	
12	124.7 CH	5.31 (1H, br s, H-12)	
13	141.4 qC		
14	41.9 qC		
15	25.5 CH_2_	1.68 (1H, m, H-15a),	
		0.98 (1H, m, H-15b)	
16	26.6 CH_2_	1.89 (1H, m, H-16a),	C-28
		1.28 (1H, m, H-16b)	
17	48.2 qC		
18	47.0 CH	2.54 (1H, m, H-18)	
19	41.6 CH_2_	2.88 (1H, t, *J* = 13.5 Hz, H-19a),	C-30
		1.97 (1H, br d, *J* = 12.0 Hz, H-19b)	
20	44.6 qC		
21	46.5 CH_2_	3.46 (1H, d, *J* = 14.5 Hz, H-21a),	C-30
		2.71 (1H, br d, *J* = 14.0 Hz, H-21b)	
22	214.9 qC		
23	28.1 CH_3_	1.32 (3H, s, Me-23)	C-3
24	15.6 CH_3_	0.86 (3H, s, Me-24)	C-3
25	16.7 CH_3_	0.91 (3H, s, Me-25)	
26	16.8 CH_3_	1.14 (3H, s, Me-26)	
27	25.5 CH_3_	1.27 (3H, s, Me-27)	C-13
28	20.9 CH_3_	1.23 (3H, s, Me-28)	C-22,C-16
29	178.6 qC		
30	21.6 CH_3_	1.42 (3H, s, Me-30)	C-29,C-19,C-21
glu			
1′	105.3 CH	5.03 (1H, d, *J* = 7.0 Hz, H-1′)	C-3
2′	83.9 CH	4.33 (1H, t, *J* = 8.0 Hz, H-2′)	C-1′,C-1″,C-4′
3′	77.7 CH	4.40 (1H, br d, *J* = 8.0 Hz, H-3′)	
4′	73.1 CH	4.64 (3H, m, H-4′, 2″, 3″)	
5′	74.7 CH	4.20 (1H, br d, *J* = 9 Hz, H-5′)	
6′	61.3 CH_2_	4.44 (2H, m, H-6′)	
glu			
1″	107.2 CH	5.26 (1H, d, *J* = 7.0 Hz, H-1″)	C-2′,C-3″
2″	74.9 CH	4.64 (3H, m, H-4′, 2″, 3″)	C-4″
3″	77.4 CH	4.64 (3H, m, H-4′, 2″, 3″)	
4″	69.5 CH	4.73 (1H, m, H-4″)	
5″	76.9 CH	4.09 (1H, t, *J* = 6.1 Hz, H-5″)	
6″	61.3 CH_2_	4.67 (2H, m, H-6″)	

All the above data identified **1** as 3-O-*β*-D-glucopyranosyl-(1→2)-*β*-D-glucopyranosyl subprogenin D. The structures of known compounds **2**–**7** were confirmed by detailed NMR data comparison with those in the literature [11,12,13,14,15].

The cytotoxicity of **1**–**7** against MCF-7, SW1990, Hela, Du-145 cancer cell lines were evaluated with 5-FU (5-Fluorouracil) and DOX (doxorubicine) as positive controls. Compound **5** showed moderate cytoxicity against SW1990, Hela, Du-145 cancer cell lines, and compound **6** showed moderate cytoxicity against MCF-7, SW1990, Du-145 cancer cell lines, whereas other compounds had no significant activity (Table 2).

**Table 2:** Cytotoxicity of **1**–**7** against four cancer cell lines.

	Cytotoxicity (IC_50_ [μg/mL])(mean ± SD%)			
	MCF-7	SW1990	Hela	Du-145
1	-^a^	-	-	-
2	-	-	-	-
3	-	-	-	-
4	-	-	-	-
5	-	5 ± 0.32	10 ± 0.89	5 ± 0.40
6	4 ± 0.18	2 ± 0.09	-	2 ± 0.08
7	-	-	-	-
DOX	1 ± 0.06		2 ± 0.16	1 ± 0.05
5-Fu		10 ± 0.95		

^a^ No significant activity at 10 μg/mL.

## 3. Experimental

### 3.1. General

1D and 2D NMR spectra were recorded on a Bruker-AV-500 spectrometer with TMS as internal standard. HRESIMS were measured with MAT 95XP mass spectrometer. IR were recorded on FT-IR Nicolet 6700. UV spectra were obtained on a Beckman DU-640 UV spectrophotometer. Optical rotations were measured with a Perkin-Elmer 341 plus. GC were run on a QP2010PLUS (Shimadzu Corporation) equipped with an ACQ mass spectrometer. For column chromatography (CC), silica gel (200–300 mesh) and GF_254_ for TLC were obtained from the Qingdao Marine Chemical Factory, Qingdao, China.

### 3.2. Plant Material

The leaves and stems of *Abrus precatorius* were collected in October 2010 from the mangrove wetlands of Hainan Island, China. The identification of the plant was performed by Professor Si Zhang. A voucher sample (No. 20101001) is maintained in the Key Laboratory of Marine Bio-Resources Sustainable Utilization, South China Sea Institute of Oceanology, Chinese Academy of Sciences, China.

### 3.3. Extraction and Isolation

The air-dried leaves and stems of *Abrus precatorius* (8 kg) were extracted with EtOH (95%, 20 L) three times (7 days each time) at room temperature. The combined extract was evaporated *in vacuo*, suspended in water, and then successively partitioned with petroleum ether, EtOAc, and *n*-butanol (800 mL × 3). The EtOAc and *n*-butanol fractions were concentrated to afford 53.7 g and 108 g of residues, resp. The EtOAc extract was subjected to silica gel CC using a gradient elution of CHCl_3_-MeOH (100:0–1:1) to afford ten fractions (Frs. **A**–**J**). Compound **3** (11 mg) was crystallizated in the bottle when eluted with the solvent CHCl_3_-MeOH 90:10 (Frs. **B**) and then purified with MeOH. Frs. **B** (7 g) was subjected to CC and eluted with CHCl_3_-acetone (30:1, 20:1, 15:1, 10:1, 5:1, each 1500 mL) to give six fractions (**B1**–**B6**), **B3** was purified by Sexphadex LH-20 (CHCl_3_-MeOH 1:1) to yield **2** (8 mg). Compound **4** (10 mg) was isolated from **B1** by repeated chromatographic on Sephadex LH-20 (CHCl_3_-MeOH 1:1) and silica gel column (CHCl_3_-MeOH 80:1). Frs. **C** (4.4 g) was subjected to CC with gradient eluting of CHCl_3_-acetone (30:1, 20:1, 15:1, 10:1, 5:1, each 1,000 mL) to give six fractions (**C1**–**C6**). Compound **5** (7 mg) was crystallizated from **C2** when eluted with the solvent CHCl_3_-acetone 20:1, and then recrystallizated with MeOH. **C4** was purified by Sexphadex LH-20 (CHCl_3_-MeOH 1:1) to give compound **6** (7 mg). Frs. **G** (2.36 g) was subjected to CC with gradient eluting of CHCl_3_-MeOH (30:1, 20:1, 15:1, 10:1, 5:1, each 500 mL) and purification on Sephadex LH-20 to yield **7** (20 mg). The *n*-BuOH extract (108 g) was subjected to CC on Amberlite XAD using MeOH-H_2_O (20%, 40%, 60% and 95%). The 40% extract part (7.23 g) was fractioned on silica gel column eluting with CHCl_3_-MeOH-H_2_O 9:1:0.1 to give ten fraction **H**–**Q**. Fraction **Q** (0.4265 g) was purified by Sexphadex LH-20 (CHCl_3_-MeOH 1:1) to give compound **1** (5 mg). Yellow powder: 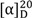 = −10 (*c* = 0.04, MOH), UV (MeOH) λ_max_ 255 nm, IR (KBr) ν_max_: 3458, 2946, 1727, 1693, 1624, 1466, 1383, 1211, 1042 cm^−^^1^; HRESI-MS *m/z* 817.4345 [M+Na]^+^ (calcd for C_42_H_66_O_14_Na, 817.4350). ^1^H and ^13^C-NMR data see Table 1.

### 3.4. Acid Hydrolysis of ***1***

Compound **1** (2 mg) was added into 3 N HCl (0.5 mL) and refluxed for 5 h in a water bath (100 °C). The solution was neutralized and extracted with EtOAc to afford the aglycon. The aglycon of **1** found to be identical with **2** by TLC. The sugars released were converted into acetylated alditols by reduction with NaBH_4_ followed by acetylation with acetic anhydride-pyridine mixture. The alditol acetates derivatives obtained were analyzed by GC using a GCMS-QP2010 Plus: The injector temperature was set at 250 °C and the column temperature program was as follows: The initial temperature of 200 °C was held constant for 2.5 min and then increased by 5 °C min to the final temperature of 250 °C. The detector temperature was set at 280 °C. MS-Scan: ACQ mode, event Time: 0.50 s with 1,000 scan speed. Alditol acetates were identified by comparison of their retention times with those of authentic samples [16]. 

### 3.5. Cytotoxicity Assays

Cytotoxicity was evaluated by the MTT [3-(4,5-dimethylthiazol-2-yl)-2,5-diphenyltetra-zolium bromide] method using MCF-7, SW1990, Hela and Du-145 cell lines. Details of the assays were described in a previous report [18].

## 4. Conclusions

The new compound 3-*O*-*β*-D-glucopyranosyl-(1→2)-*β*-D-glucopyranosyl subprogenin D (**1**) was isolated from *Abrus precatorius*, together with six known triterpenoids. Compounds **5** and **6** showed moderate cytoxicities against MCF-7, SW1990, Hela and Du-145. However, the new compound **1** and the other known ones had no significant activity.

## References

[1] Ma C.M., Nakamura N., Hattori M. (1998). Saponins and *C*-glycosyl flavones from the seeds of *Abrus precatorius*. Chem. Pharm. Bull..

[2] Dnyaneshwar J.T., Ravindra Y.P. (2012). Effect of *Abrus precatorius* leaves on milk induced leukocytosis and eosinophilia in the management of asthma. Asian Pac. J. Trop. Med..

[3] Ghosal S., Dutta S.K. (1971). Alkaloids of *Abrus precatorius*. Phytochemistry.

[4] Markham K.R., Wallace J.W., Babu Y.N., Murty V.K., Rao M.G. (1989). 8-*C*-Glucosylscutel larein 6,7-dimethyl ether and its 2″-*O*-apioside from *Abrus precatorius*. Phytochemistry.

[5] Namcheol K., Darrick S.H.L.K., Kinghorn D.A. (2001). New triterpenoids from the leaves of *Abrus precatorius*. Nat. Prod. Lett..

[6] Qing S.C., Hu Z.B. (1998). Abruquinone A, B, D, E, F and G from the root of *Abrus precatorius*. Acta Bot. Sin..

[7] Anam E.M. (2001). Anti-inflammatory activity of compounds isolated from the aerial parts of *Abrus precatorius (Fabaceae)*. Phytomedicine.

[8] Yadava R.N., Sudhan Reddy V.M. (2002). A new biological activity flavonol glycoside from the seeds of *Abrus precatorius linn*. J. Asian. Nat. Prod. Res..

[9] Kuo S.C., Chen S.C., Chen L.H., Wu J.B., Wang J.P., Teng C.M. (1995). Potent antiplatelet, anti-inflammatory and antiallergic isoflavanquinones from the roots of *Abrus precatorius*. Planta Med..

[10] Dimetry N.Z., El-Gengaihi S., Reda A.S., Amer S.A.A. (1992). Biological effects of some isolated *Abrus precatorius* L. alkaloids towards *Tetranychus urticae* Koch. J. Pest. Sci..

[11] Takeshito T., Yokoyama K., Ding Y., Kinjo J., Nohara T. (1991). Four new and twelve known sapogenols from *Sophorae subprostratae radix*. Chem. Pharm. Bull..

[12] Chiang T.C., Chang H.M., Mak T.C. (1983). New oleanene-type triterpenes from *Abrus precatorius* and X-ray crystal structure of abrusgenic acid-methanol 1:1 solvate. Planta Med..

[13] Zhang C.P., Zhang Y.G., Lv X.Y., Chen Y., Ma P.C., He C.H., Yu D.Q., Shen F.L., Yang J.J., Yang J. (1989). Studes on triterpenoids of total glucosides of *Tripterygium wilfordii* (TII). Acta Acad. Med. Sin..

[14] Chang H.M., Chiang T.C., Thomas C.W.M. (1982). Isolation and structure elucidation of abruslactone A: A new oleanene-type triterpene from the roots and vines of *Abrus precatorius* L. J. Chem. Soc. Chem. Commun..

[15] Choi Y.H., Hussain R.A., Pezzuto J.M., Kinghorn A.D., Morton J.F. (1989). Abrusosides A–D, four novel sweet-tasting triterpene glycosides from the leaves of *Abrus precatorius*. J. Nat. Prod..

[16] De Rosa S., Iodice C., Mitova M., Handjieva N., Popov S., Anchev M. (2000). Triterpene saponins and iridoid glucosides from *Galium rivale*. Phytochemistry.

[17] Debellaa A., Haslingera E., Schmida M.G., Bucard F., Michlb G., Abebec D., Kunert O. (2000). Triterpenoid saponins and sapogenin lactones from *Albizia Gummifera*. Phytochemistry.

[18] Wang F.Z., Tian X.P., Huang C.G., Li Q.X., Zhang S. (2011). Marinactinones A–C, new γ-pyrones from marine actinomycete *Marinactinospora thermotolerans* SCSIO 00606. J. Antibiot..

